# Effects of Dietary Inclusion of Perilla Seed Meal on Growth Performance, Plasma Biochemistry, and Breast Muscle Fatty Acid Composition in *Sansui* Ducks from 4 to 8 Weeks of Age

**DOI:** 10.3390/ani16060860

**Published:** 2026-03-10

**Authors:** Yulong Feng, Meijuan Li, Chunpei Yang, Shunbo Yu, Yuxi Lu, Yongbao Wu, Zhiguo Wen

**Affiliations:** 1 Institute of Animal Husbandry and Veterinary Medicine, Guizhou Academy of Agricultural Sciences, Guiyang 550005, China; keshili.happy@163.com (M.L.); 17385366041@163.com (Y.L.); 2 Sansui Bureau of Agriculture and Rural Affairs, Qiandongnan Prefecture 556500, China; yangchunpei88@163.com; 3 Guizhou Youyan Chunxiang Ecological Grain and Oil Technology Co., Ltd., Guiyang 550005, China; shby2000@163.com; 4 Key Laboratory of Feed Biotechnology of Ministry of Agriculture and Rural Affairs, Institute of Feed Research, Chinese Academy of Agricultural Sciences, Beijing 100081, China; wuyongbao@caas.cn

**Keywords:** perilla seed meal, Sansui ducks, growth performance, fatty acids, plasma biochemistry

## Abstract

This study evaluated the effects of dietary perilla seed meal (PSM) inclusion on growth performance, plasma biochemistry, and breast muscle fatty acid composition in Sansui ducks (Sansui Sheldrake ducks) from 4 to 8 wks of age. A total of 320 male ducks, 29 d old, were randomly assigned to 5 dietary treatments (0, 5%, 10%, 15%, or 20% PSM) with 8 replicates each group and 8 ducks per replicate. Results showed that PSM inclusion up to 20% did not significantly (*p* > 0.05) affect body weight, daily gain, feed intake, feed-to-gain ratio, or carcass traits. Plasma albumin, total cholesterol, and high-density lipoprotein cholesterol were elevated at 10% PSM inclusion (*p* < 0.05), while liver function markers remained unaffected (*p* > 0.05). As expected, dietary PSM supplementation dose-dependently enriched breast muscle n-3 polyunsaturated fatty acids, including α-linolenic acid, eicosapentaenoic acid, and docosahexaenoic acid (*p* < 0.05), and reduced the n-6/n-3 ratio (*p* < 0.05). These findings indicated that PSM could be incorporated into duck diets at levels up to 20% to enhance the nutritional value of duck meat without adverse effects on growth performance and health, supporting its use as a functional feed ingredient in sustainable duck production.

## Simple Summary

The poultry industry has been increasingly seeking sustainable and cost-effective protein sources for animal feed. Perilla seed meal (PSM), a by-product of oil extraction, is rich in protein and beneficial fatty acids, making it a promising alternative that can partially replace conventional ingredients like soybean meal. In this study, we investigated the effects of including different levels of PSM (0, 5%, 10%, 15%, and 20%) in the diets of Sansui ducks from 4 to 8 weeks of age. We found that even at the highest inclusion level (20%), the ducks’ growth and body composition remained unchanged. However, the fatty acid profile of their breast meat was significantly improved, with higher levels of health-promoting n-3 fatty acids. These results suggested that PSM could be safely used in duck feed to enhance the nutritional quality of duck meat without compromising growth performance, supporting the development of more sustainable poultry production systems.

## 1. Introduction

The poultry industry is actively seeking sustainable and economical alternatives to conventional protein sources like soybean meal and fishmeal. This drive is motivated by the rising costs of these ingredients and the significant environmental burdens of their production, such as land-use change, high water consumption, and greenhouse gas emissions [1,2]. Among the promising alternatives, plant-derived protein meals from oilseed crops, such as rapeseed meal (RSM) and perilla seed meal (PSM), have gained significant attention due to their broad availability, balanced nutritional composition, and potential to reduce feed costs [3,4,5]. Furthermore, the utilization of these plant-based proteins not only helps alleviate competition between human and animal feed uses but also promotes the valorization of agricultural by-products, aligning with the strategic needs of sustainable livestock and poultry production. 

*Perilla frutescens*, an annual crop belonging to the Lamiaceae (Labiatae) family, is commonly known as perilla. Its leaves and seeds represent the primary applicable parts of the plant. Notably, perilla seeds are rich in polyphenols and flavones and serve as a valuable source of omega-3 polyunsaturated fatty acids (PUFAs), particularly alpha-linolenic acid (ALA), for oil production. PSM, a by-product of Perilla seed oil extraction, is characterized by its high crude protein content (31.54% to 43.2%) [6,7,8], abundant n-3 polyunsaturated fatty acids (PUFA), notably α-linolenic acid (ALA) and linoleic acid (LA), and a range of bioactive compounds [9,10,11,12,13]. It also contains essential minerals such as phosphorus, calcium, iron, zinc, etc. [13]. Nevertheless, PSM has a notably high crude fiber content (18.8% to 24.43%) [6,8]. These nutritional properties position PSM as a promising ingredient for poultry diets, particularly for enhancing the n-3 fatty acid content of meat and eggs, thereby aligning with increasing consumer demand for healthier animal products [12,14]. However, the presence of high crude fiber content may restrict its dietary inclusion level, underscoring the need for further research to optimize its application in poultry nutrition [13]. Existing studies on the dietary inclusion of PSM in poultry have yielded inconsistent outcomes. Oh et al. observed that partial substitution of soybean meal with 2% PSM in broiler diets over a 5-week period (1 to 35 days) significantly improved growth performance, meat quality, and fatty acid composition [8]. In contrast, supplementation with 10% PSM in Korean native chickens over a 20-day trial showed no statistically significant differences in growth parameters or relative visceral organ weights (including liver, abdominal fat, and breast muscle) compared with the non-supplemented group, although ALA content in breast muscle was markedly increased relative to basal diet controls [15]. Similarly, in a 42-day trial involving broilers, incremental PSM inclusion (2–8%) led to a dose-dependent reduction in the n-6/n-3 PUFA ratio in both breast and thigh meat [12]. It is worth noting, however, that current research remains largely concentrated on broilers, with limited empirical evidence available regarding the effects of PSM in ducks. 

The Sansui Sheldrake duck, an indigenous breed from Guizhou Province, China, exhibits a high demand for dietary protein to achieve its genetic potential for rapid growth and efficient feed utilization [16,17]. This study aimed to evaluate the impact of incremental dietary PSM levels on growth performance, plasma biochemical parameters, and breast muscle fatty acid composition in Sansui ducks (4–8 weeks), thereby assessing its feasibility as a sustainable alternative protein source. The results are expected to furnish essential evidence for integrating PSM into duck diets, which would support sustainable poultry nutrition and advance the eco-friendly utilization of agricultural by-products.

## 2. Materials and Methods

### 2.1. Perilla Seed Meal

PSM was sourced from Guizhou Youyan Chunxiang Ecological Grain and Oil Technology Co., Ltd. (Guiyang, China). The proximate composition of PSM (dry matter basis) was 41.32% crude protein (CP) and 13.62% crude fiber (CF). The CP content was determined via the Dumas combustion method (AOAC) [18], while CF was analyzed following the procedure of the National Standards Committee (2022) [19]. The essential amino acids and main fatty acids of perilla seed meal were shown in Table 1, Tables S1 and S2. Amino acid contents were determined following the detailed procedures of which have been described in our previous report [17]. Briefly, Amino acids were hydrolyzed with 6 M HCl at 110 °C for 24 h, except for methionine and cystine, which were oxidized with performic acid (9:1 formic acid: H_2_O_2_) for 16 h prior to hydrolysis under the same conditions. Afterwards, amino acid quantitation in diets were determined using the Hitachi L-8800 Amino Acid Analyzer (Tokyo, Japan). The apparent metabolizable energy (AME, 14.02 MJ/kg) and true metabolizable energy (TME, 16.38 MJ/kg), as well as amino acid digestibility for Sansui ducks (Table S3), were determined using the emptying-force feeding method, as reported by Wei et al. [20]. 

### 2.2. Birds Management and Diets

All procedures were approved by the Institute of Animal Husbandry and Veterinary Medicine, Guizhou Academy of Agricultural Sciences (Grant No.202405/2024-05). Sansui ducklings from a commercial hatchery (Guizhou Sansui Duck Industry Operation and Development Co., Ltd., Guizhou, China) were raised from hatch to 4 wk of age with common starter diets (AME, 11.72 MJ/kg; CP, 20.0%) under ad libitum feeding. Subsequently, a total of 320 birds (body weight, 559.5 ± 28.1 g) were selected at 29 d of age, and randomly allocated to 5 groups with 8 replicates each group and 8 ducks per replicate. The ducks were raised in plastic-floor pens (200 × 100 × 60 cm) from 4 to 8 wk of age. During the 4-wk trial, all ducks had ad libitum access to feed and water. The ambient temperature was maintained at 33 °C (1 to 3 d of age), gradually reduced to 25 °C by 21 d of age, and then stabilized at 24 to 26 °C. Relative humidity was maintained at 20% from 1 to 3 days of age, after which it was gradually increased and subsequently stabilized at approximately 65%.

The experimental diets were formulated to contain graded inclusion levels of PSM at 0%, 5%, 10%, 15%, or 20% (Table 2). All diets were isoenergetic (AME, 12 MJ/kg) and isonitrogenous (CP, 17%), meeting or exceeding the nutrient requirements recommended by the Ministry of Agriculture of China for ducks during the grower-finisher period [21]. After mixing, all diets were prepared in mash form and subsequently cold-pelleted at room temperature.

### 2.3. Data and Sample Collection

At the conclusion of the 8-week trial, all ducks were fasted for 12 h. All ducks within each pen were weighed to determine the average body weight. Feed consumption was also recorded on a pen basis, from which the average daily gain (ADG), average daily feed intake (ADFI), and feed conversion ratio (FCR) were calculated. Subsequently, two ducks per pen with body weights closest to the average body weight were selected for sampling. Before slaughtering, blood samples were collected from the brachial vein of these ducks into heparinized tubes. The blood samples were centrifuged at 3500× *g* for 15 min at 4 °C to obtain plasma, which was then stored at −20 °C for subsequent analysis. The selected ducks were then euthanized by CO_2_ inhalation and exsanguinated. The liver, breast muscle, thigh muscle, and abdominal fat (including fat surrounding the proventriculus, gizzard, abdominal wall, and cloaca) were immediately excised and weighed. Their weights were expressed as a percentage of the live body weight. Approximately 2 g of breast muscle sample were collected and stored at −80 °C for fatty acid analysis. 

### 2.4. Plasma Parameters Analyses

Plasma biochemical parameters were determined spectrophotometrically using an automatic biochemical analyzer (Hitachi 7080, Tokyo, Japan) with corresponding commercial assay kits (Maccura, Chengdu City, China). The analyzed profile encompassed alanine aminotransferase (ALT), aspartate aminotransferase (AST), alkaline phosphatase (ALP), total protein (TP), albumin (ALB), uric acid (UA), triglyceride (TG), total cholesterol (CHO), high-density lipoprotein cholesterol (HDL-C), and low-density lipoprotein cholesterol (LDL-C). The globulin (GLB) concentration was calculated as the difference between TP and ALB.

### 2.5. Fatty Acids Analyses

Breast muscle and PSM samples were freeze-dried, ground, and analyzed for their fatty acid composition. The analysis was performed using a Trace 1300 gas chromatograph coupled to an ISQ 7000 mass spectrometer (Thermo Fisher Scientific, Waltham, MA, USA). The GC was equipped with a Thermo TG-FAME capillary column (50 m × 250 µm × 0.20 µm). Helium served as the carrier gas at a flow rate of 0.63 mL/min. Samples were injected in split mode (8:1) with a 1 μL volume at an injector temperature of 250 °C. The column temperature program was: 80 °C (1 min), increased to 160 °C at 20 °C/min (held for 1.5 min), then to 196 °C at 3 °C/min (held for 8.5 min), and finally to 250 °C at 20 °C/min (held for 3 min). The ion source and transfer line temperatures were 300 °C and 280 °C, respectively. Mass spectra were acquired in Scan/SIM mode (*m*/*z* 33–400) with a 7 min solvent delay using electron impact ionization at −70 eV. Fatty acids were identified by matching retention times and mass spectra with commercial standards (Anpel Laboratory Technologies Inc., Shanghai, China) and quantified based on peak areas. Results are expressed as mg per gram of sample (dry matter basis). 

### 2.6. Statistical Analysis

Following confirmation of homogeneity and normality (Shapiro–Wilk test), data were analyzed by one-way ANOVA using SAS 9.0 [22]. When a significant treatment effect was detected (*p* < 0.05), means were compared using Duncan’s multiple range test. The replicate pen was considered the experimental unit for all analyses. Variability within the data is presented as the pooled standard error of the mean (SEM).

## 3. Results

### 3.1. Growth Performance and Carcass Traits

Compared with the control (0% PSM), dietary PSM inclusion did not significantly (*p* > 0.05) affect the growth performance, of Sansui ducks from 4 to 8 wk of age, as measured by BW, ADG, ADFI, and FCR (Table 3). Similarly, PSM supplementation did not alter the carcass traits at 8 wk of age, including breast muscle yield, thigh muscle yield, and the relative weights of the liver and abdominal fat (Table 4).

### 3.2. Plasma Biochemical Parameters

As presented in Table 5, dietary PSM inclusion significantly (*p* < 0.05) altered the plasma concentrations of ALB, CHO, HDL-C, and LDL-C, whereas no significant changes (*p* > 0.05) were observed in ALT, AST, ALP, TP, GLB, UA, or TG. The plasma albumin (ALB) concentration increased linearly (*p* < 0.05) with increasing dietary PSM and plateaued at the 10% inclusion level. Compared to the control (0% PSM) diet, only the 10% PSM inclusion resulted in a significant elevation (*p* < 0.05) in plasma concentrations of CHO, HDL-C, and LDL-C; other inclusion levels did not produce this effect.

### 3.3. Breast Muscle Fatty Acid Composition

As shown in Table 6, dietary supplementation with PSM significantly modified the breast muscle fatty acids profile of Sansui ducks, especially the n-3 PUFAs. Increasing dietary PSM from 0% to 20% induced a dose-dependent enrichment of n-3 PUFAs. The content of α-linolenic acid (ALA, C18:3 n-3) increased significantly from 7.07 to 50.78 μg/g (*p* < 0.05), while the long-chain derivatives eicosapentaenoic acid (EPA), docosapentaenoic acid (DPA), and docosahexaenoic acid (DHA) increased by 6.3-fold, 2.5-fold, and 2.6-fold, respectively (all *p* < 0.05). Meanwhile, the n-6 PUFAs showed a numerical but non-significant decrease with increasing dietary PSM level (*p* > 0.05), although C20:4 n-6, C22:4 n-6, and C22:5 n-6 were significantly affected (*p* < 0.05). Expectedly, the n-6/n-3 PUFA ratio progressively decreased from 13.47 to 3.40 (*p* < 0.05). The predominant fatty acids in breast muscle were C16:0, C18:0, C18:1 n-9, C18:2 n-6, and C20:4 n-6. Among these, only the concentration of C20:4 n-6 (arachidonic acid, AA) was significantly altered by dietary PSM inclusion (*p* < 0.05), while the others remained unaffected. While total saturated fatty acids (SFA) and total monounsaturated fatty acids (MUFA) remained stable, targeted changes occurred in specific low-abundance SFAs (C6:0, C11:0, C12:0, C23:0) and MUFAs (C17:1, C18:1 n-12, C20:1) (*p* < 0.05). 

## 4. Discussion

In the present study, the dietary inclusion of PSM at 5%, 10%, 15%, and 20% in Sansui ducks from 4 to 8 wk of age neither affected growth performance (BW, ADG, ADFI, and FCR) nor altered the carcass traits measured at 8 wk. Previously, research on the application of perilla seed meal (PSM) in poultry diets remains limited. Consistent with our results, Zhang et al. investigated the effect of perilla seed meal supplementation (0.3–1%) in breeding hen diets and reported that while average egg weight, laying rate, and feed conversion ratio (FCR) remained unchanged, the fertilization rate and hatchability were significantly enhanced [14]. Peiretti et al. found that perilla seed meal supplementation (5% and 10%) in isonitrogenous and isocaloric rabbit diets had no significant effects on ADFI, ADG, and FCR [23]. Hadi et al. reported that supplementing diets with up to 5% *Perilla frutescens* seeds (25.01% CP, 29.93% CF) significantly increased the average daily gain (ADG) of local ducks, while no differences were observed among groups in terms of feed intake, slaughter weight, or carcass weight [24]. On the contrary, in a study where perilla seed meal (PSM) was used to incrementally replace soybean meal (0.5%, 1%, and 2%) in broiler diets from 0 to 5 wk of age, Han et al. found that the 1% and 2% PSM inclusion levels significantly improved final body weight and weight gain compared to the control and 0.5% groups. Specifically, the 2% PSM inclusion reduced feed intake, whereas the 1% inclusion level optimized feed conversion efficiency, yielding the lowest feed conversion ratio among all dietary treatments [7]. This discrepancy may be attributable to the age and feeding duration of the animals, as well as to potential variations in their physiological sensitivity to the bioactive compounds present in PSM. Taken together, these results indicate that replacing soybean meal with perilla seed meal (up to 20%) had no negative effects on growth.

Furthermore, we assessed its physiological effects by analyzing a panel of plasma biochemical indices. As expected, the inclusion of PSM in the diet did not elevate plasma activities of the hepatic enzymes ALT, ALP, or AST—biomarkers indicative of hepatocellular integrity in poultry [25]. Combined with the finding that the relative liver weight remained stable across treatment groups, this suggested that PSM supplementation did not induce hepatic damage and was associated with a state of metabolic homeostasis. The linear rise in plasma albumin (ALB) with increasing PSM inclusion is consistent with its balanced amino acid composition (Table S1), which could enhance hepatic protein synthesis and subsequent albumin release into circulation [26]. A significant increase in plasma CHO, HDL-C, and LDL-C was observed specifically at the 10% PSM inclusion level. These outcomes align with the findings of Hadi et al. [27] perilla seed supplementation increased cholesterol content in duck breast meat. In contrast, several studies have reported divergent physiological responses in terms of perilla seed oil. Cui et al. [28] observed that 1% dietary perilla seed oil notably lowered plasma CHO, and LDL-C levels in chickens, while HDL-C remained unaffected. Pothinam et al. [29] documented that perilla seed oil significantly reduced blood CHO by 17.16% and 15.91% in hyperlipidemic rats fed a high-fat or normal diet, respectively. In contrast, plasma levels of HDL-C and LDL-C were not significantly altered by the treatment. These discrepancies probably stemmed from differences in the chemical composition of the perilla-derived ingredients used across studies [30]. 

The high contents of essential fatty acids (LA and ALA) in PSM provided a dietary basis for significantly altering the breast muscle fatty acid profile in Sansui ducks, owing to the fact that birds lack the enzymatic capacity for their de novo synthesis [31]. Specifically, we observed a clear dose-dependent enrichment of n-3 PUFAs (ALA, DPA, DHA) coupled with a decrease in specific long-chain n-6 PUFAs (C20:4, C22:4, C22:5 n-6), resulting in a markedly reduced n-6/n-3 ratio. This selective remodeling mechanistically explained by the competitive metabolism of LA and ALA in the liver was also observed in rabbits fed diet supplemented with PSM [23], laying hens fed rubber seed oil [32], and ducks fed flaxseed diet [33]. In the liver, LA and ALA serve as precursors for the n-6 and n-3 PUFAs, respectively. They undergo parallel transformation via a series of desaturation (catalyzed by Δ-6 and Δ-5 desaturases) and elongation reactions. Specifically, ALA is sequentially converted to EPA, then to DPA, and finally to DHA. Conversely, LA is metabolized into a suite of n-6 derivatives, including C18:3 n-6, C20:3 n-6, C20:4 n-6, C22:4 n-6, and C22:5 n-6 [34,35]. Consequently, the elevated n-3 PUFA content and reduced n-6/n-3 ratio enhance the nutritional quality of the meat. This nutritional profile is associated with benefits for lipid metabolism and cardiovascular health [36], which aligns with growing consumer demand for functionally enriched foods, thereby increasing the market potential of duck meat produced under this feeding strategy. A key limitation of this study is the absence of quantitative data on the active components of PSM, as well as the lack of investigation into the underlying physiological mechanisms. Consequently, although our findings support the practical application of PSM in feed, further research is necessary to characterize its bioactive constituents and elucidate their regulatory effects on animal health.

## 5. Conclusions

In conclusion, our findings collectively demonstrate that dietary PSM inclusion of up to 20% safely improves lipid metabolism and increases n-3 PUFA deposition in Sansui duck breast muscle, while maintaining growth performance and carcass traits. The strategic use of PSM thereby represents an effective approach to simultaneously enhance the nutritional value of duck meat and support metabolic health, providing a theoretical basis for developing functional waterfowl products. 

## Figures and Tables

**Table 1:** The essential amino acids and main fatty acids of perilla seed meal (as-fed basis).

Essential Amino Acids	Content (%)	Fatty Acids	Content (μg/g)
Methionine	0.902	C16:0	785.04
Cysteine	0.628	C16:1	21.00
Lysine	1.380	C18:0	222.20
Threonine	1.215	C18:1 n-9	791.89
Arginine	3.597	C18:1 n-12	1181.75
Isoleucine	1.183	C18:2 n-6 (LA)	2071.86
Leucine	2.159	C18:3 n-3 (ALA)	8750.80
Valine	1.563	C20:1	3510.09
Phenylalanine	1.694	C20:3 n-3	8.80
		C20:4 n-6 (AA)	4.50
		C20:5 n-3 (EPA)	2.66

**Table 2:** Composition and nutrient levels in experimental diets of Sansui ducks from 4 to 8 wk of age (as-fed basis).

Ingredient (%)	Dietary Perilla Seed Meal Level (%)				
	0	5	10	15	20
Corn	60.20	58.00	57.00	55.50	54.50
Corn starch	3.70	4.30	4.65	4.65	4.72
Rice hull powder	4.27	5.27	5.80	6.83	7.51
Soybean meal	26.71	22.35	17.75	13.25	8.65
Perilla Seed Meal	0.00	5.00	10.00	15.00	20.00
Soybean oil	1.03	1.00	0.70	0.66	0.47
Sodium chloride	0.30	0.30	0.30	0.30	0.30
Dicalcium phosphate	1.54	1.50	1.50	1.50	1.50
Limestone	1.10	1.10	1.07	1.05	1.05
Vitamin and mineral premix ^1^	1.00	1.00	1.00	1.00	1.00
*DL*-Methionine	0.14	0.13	0.13	0.12	0.12
*L*-Lysine sulphate	0.01	0.05	0.10	0.14	0.18
Total	100.00	100.00	100.00	100.00	100.00
Calculated Nutrient Levels					
AME (MJ/kg) ^2^	12.00	12.00	12.00	12.00	12.00
Crude protein (%)	17.00	17.00	17.00	17.00	17.00
Calcium (%)	0.86	0.86	0.85	0.86	0.86
Available phosphorus (%)	0.38	0.38	0.38	0.38	0.38
Methionine (%)	0.41	0.41	0.41	0.41	0.41
Methionine + Cysteine (%)	0.71	0.71	0.70	0.70	0.70
Lysine (%)	0.86	0.86	0.86	0.86	0.86

^1^ Supplied per kilogram of total diet: Fe (FeSO_4_·7H_2_O), 60 mg; Cu (CuSO_4_·5H_2_O), 10 mg; Mn (MnSO_4_·H_2_O), 100 mg; I (KI), 0.4 mg; Zn (ZnO), 60 mg; Se (NaSeO_3_), 0.3 mg; vitamin A (Retinol acetate), 10,000 IU; vitamin D_3_ (Cholcalciferol), 3000 IU; vitamin E (*DL-*α-tocopheryl acetate), 20 IU; vitamin K_3_ (menadione sodium bisulfate), 2.5 mg; riboflavin, 15 mg; thiamin (thiamin mononitrate), 2 mg; pyridoxine hydrochloride, 4 mg; calcium-*D*-pantothenate, 20 mg; cobalamin, 0.06 mg; nicotinic acid, 50 mg; folic acid, 1 mg; biotin, 0.2 mg; choline chloride, 1500 mg. ^2^ The AME values of the experimental diets were derived from the AME values of individual feedstuffs for ducks.

**Table 3:** Effects of dietary PSM on growth performance of Sansui ducks from 4 to 8 wk of age ^1^.

Dietary PSM Level, %	BW, g	ADG, g/d/Bird	ADFI, g/d/Bird	FCR, g/g
0	1123.13	20.75	119.33	5.78
5	1119.54	20.54	118.17	5.76
10	1113.75	20.55	118.60	5.80
15	1110.00	20.53	119.39	5.85
20	1111.11	20.53	119.13	5.83
SEM	5.00	0.21	0.25	0.06
*p*-Value	0.9173	0.9974	0.4719	0.9927

^1^ Values are mean of 8 replicates of 8 ducks. BW, body weight; ADFI, average daily feed intake; ADG, average daily gain; FCR, feed conversion ratio.

**Table 4:** Effects of dietary PSM on carcass traits of Sansui ducks at 8 wk of age ^1^.

Dietary PSM Level, %	Breast Muscle, %	Thigh Muscle, %	Liver, %	Abdominal Fat, %
0	6.585	9.478	2.161	0.561
5	5.665	8.751	2.278	0.593
10	6.226	10.180	2.218	0.350
15	5.626	8.571	2.243	0.444
20	6.306	10.328	2.368	0.574
SEM	0.236	0.437	0.038	0.058
*p*-Value	0.6586	0.6327	0.5267	0.6797

^1^ Values are mean of 8 replicates of 2 ducks.

**Table 5:** Effects of dietary PSM on plasma chemical index of Sansui ducks aged 4 to 8 wks ^1^.

Items	Dietary PSM Level (%)					SEM	*p*-Value
	0	5	10	15	20		
ALT, U/L	38.54	42.67	33.09	39.1	31.47	1.77	0.2497
AST, U/L	37.63	44.51	38.59	42.59	34.27	2.08	0.5660
ALP, U/L	537.69	554.1	546.08	467.55	539.97	20.05	0.6774
TP, g/L	41.43	41.16	46.71	42.27	38.83	1.09	0.2353
ALB, g/L	20.3 ^c^	22.04 ^bc^	24.29 ^a^	22.69 ^ab^	21.44 ^bc^	0.34	0.0012
GLB, g/L	21.13	19.12	22.43	19.58	17.39	0.89	0.4574
UA, μmol/L	104.12	87.33	112.14	87.95	119.14	9.83	0.8112
TG, μmol/L	0.69	0.72	0.82	0.68	0.66	0.02	0.0949
CHO, μmol/L	5.87 ^b^	5.84 ^b^	7.27 ^a^	6.39 ^b^	6.28 ^b^	0.14	0.0040
HDL-C, μmol/L	3.09 ^b^	2.98 ^b^	3.71 ^a^	3.26 ^b^	3.27 ^b^	0.07	0.0160
LDL-C, μmol/L	1.69 ^b^	2.02 ^ab^	2.22 ^a^	2.04 ^ab^	1.77 ^b^	0.06	0.0166

^1^ Values are mean of 8 replicates of 2 ducks. ^a–c^ Within a row, means without a common superscript differed (*p* < 0.05). ALT, alanine aminotransferase; AST, aspartate aminotransferase; ALP, alkaline phosphatase; TP, total protein; ALB, albumin; GLB, globulin; UA, uric acid; TG, triglyceride; CHO, total cholesterol; HDL-C, high-density lipoprotein cholesterol; LDL-C, low-density lipoprotein cholesterol.

**Table 6:** Effects of dietary PSM on breast muscle fatty acids profile of Sansui ducks aged 4 to 8 wks ^1^.

Fatty Acids, μg/g	Dietary PSM Level (%)					SEM	*p*-Value
	0	5	10	15	20		
C6:0	0.398 ^a^	0.276 ^bc^	0.385 ^ab^	0.293 ^abc^	0.254 ^c^	0.019	0.0272
C8:0	0.208	0.174	0.186	0.173	0.159	0.007	0.2170
C10:0	0.176	0.196	0.210	0.179	0.178	0.014	0.9253
C11:0	0.128 ^a^	0.128 ^a^	0.125 ^a^	0.121 ^a^	0.106 ^b^	0.002	0.0020
C12:0	1.470 ^a^	1.026 ^ab^	0.691 ^b^	0.556 ^b^	0.554 ^b^	0.099	0.0042
C13:0	0.134	0.138	0.140	0.131	0.131	0.002	0.6143
C14:0	3.640	4.314	5.319	4.006	4.371	0.565	0.9097
C14:1	0.371	0.414	0.524	0.400	0.436	0.037	0.7345
C15:0	0.751	0.814	0.901	0.740	0.798	0.060	0.9231
C15:1	1.368	0.540	0.639	0.619	0.678	0.107	0.0640
C16:0	443.069	490.702	453.783	370.886	446.133	31.764	0.8724
C16:1	13.996	15.696	19.575	15.664	17.935	2.415	0.9566
C17:0	1.953	2.178	2.286	1.990	2.354	0.138	0.8576
C17:1	19.845 ^a^	14.264 ^b^	14.27 ^b^	14.197 ^b^	14.181 ^b^	0.605	0.0014
C18:0	383.285	387.326	339.47	309.219	393.005	20.797	0.6777
C18:1 n-12	63.874 ^ab^	80.570 ^a^	61.123 ^ab^	44.876 ^bc^	29.554 ^c^	4.824	0.0092
C18:1 n-9	437.484	482.382	490.953	423.727	477.604	51.862	0.9931
C18:1 n-7	51.410	45.538	43.435	36.276	56.289	4.112	0.5983
C18:2 n-6, LA	323.218	316.67	336.905	277.953	354.363	25.903	0.9226
C20:0	3.973	4.346	4.690	3.360	4.938	0.413	0.7806
C18:3 n-6	0.703	0.732	0.728	0.589	0.745	0.049	0.8705
C20:1	3.949 ^b^	17.510 ^ab^	21.000 ^ab^	20.933 ^ab^	32.244 ^a^	3.371	0.0419
C18:3 n-3, ALA	7.071 ^b^	26.54 ^ab^	34.684 ^ab^	48.769 ^a^	50.784 ^a^	4.903	0.0135
C21:0	0.348	0.396	0.366	0.341	0.381	0.018	0.8947
C20:2	5.419	5.646	5.373	4.326	5.555	0.301	0.6835
C22:0	2.998	2.820	2.988	2.179	3.496	0.272	0.6695
C20:3 n-6	12.111	12.688	10.566	9.983	13.109	0.784	0.6872
C22:1 n-9	1.385	1.310	1.191	1.329	1.733	0.082	0.2526
C20:3 n-3	0.609 ^b^	0.938 ^b^	1.334 ^b^	1.484 ^b^	2.689 ^a^	0.203	0.005
C20:4 n-6, AA	321.523 ^a^	333.614 ^a^	264.121 ^ab^	215.601 ^b^	254.066 ^ab^	13.988	0.0489
C23:0	0.163 ^b^	0.246 ^b^	0.294 ^ab^	0.409 ^ab^	0.609 ^a^	0.052	0.0376
C22:2	0.459	0.444	0.368	0.374	0.416	0.017	0.331
C20:5 n-3, EPA	2.869 ^b^	4.66 ^b^	9.014 ^b^	8.673 ^b^	18.008 ^a^	1.273	0.0001
C24:0	0.361	0.722	0.92	0.807	1.444	0.147	0.1786
C24:1	0.671	0.678	0.545	0.509	1.076	0.117	0.5385
C22:4 n-6	49.221 ^a^	46.13 ^a^	29.713 ^b^	26.039 ^b^	32.154 ^b^	2.252	0.0004
C22:5 n-6	51.025 ^a^	35.422 ^b^	17.911^c^	17.756 ^c^	18.190 ^c^	2.648	<0.0001
C22:5 n-3, DPA	19.221 ^b^	29.862 ^b^	29.535^b^	31.064 ^b^	47.363 ^a^	2.765	0.0102
C22:6 n-3, DHA	26.999 ^c^	46.868 ^b^	50.064^b^	57.874^ab^	71.386 ^a^	3.683	0.0003
SFA	843.048	895.804	812.753	695.376	858.901	51.045	0.8199
MUFA	594.349	658.894	653.246	558.523	631.725	62.044	0.9898
PUFA	820.445	860.204	790.306	700.483	868.815	44.336	0.7917
PUFA/SFA	0.974	0.996	1.014	1.023	1.028	0.019	0.8912
n-3 PUFA	56.773 ^c^	108.866 ^bc^	124.629 ^b^	147.866 ^ab^	190.225 ^a^	11.359	0.0005
n-6 PUFA	708.575	699.122	630.228	521.88	640.465	36.489	0.5466
n-6/n-3	13.465 ^a^	6.648^b^	5.099 ^bc^	3.583^c^	3.396^c^	0.71	<0.0001

^1^ Values are mean of 8 replicates of 2 ducks. ^a–c^ Within a row, means without a common superscript differed (*p* < 0.05). LA, linoleic acid; ALA, α-linolenic acid; AA, arachidonic acid; EPA, eicosapentaenoic acid; DPA, docosapentaenoic acid; DHA, docosahexaenoic acid; SFA, saturated fatty acid; MUFA, monounsaturated fatty acid; PUFA, polyunsaturated fatty acid.

## Data Availability

The data presented in this study are available on request from the corresponding author on reasonable request.

## Supplementary Materials

The following supporting information can be downloaded at: https://www.mdpi.com/article/10.3390/ani16060860/s1, Table S1: The amino acid composition of perilla seed meal (as-fed basis); Table S2: The fatty acid composition of perilla seed meal (as-fed basis); Table S3: The amino acid digestibility of perilla seed meal on Sansui ducks by emptying-force feeding method.

## Abbreviations

The following abbreviations are used in this manuscript:

ALA	α-linolenic acid
ALB	albumin
ALP	alkaline phos-phatase
ALT	alanine aminotransferase
AME	apparent metabolizable energy
AMPK	AMP-activated protein kinase
AST	aspartate aminotransferase
CHO	total cholesterol
DHA	docosahexaenoic acid
DPA	docosapentaenoic acid
EPA	eicosapentaenoic acid
GLB	globulin
HDL-C	high-density lipoprotein cholesterol
LA	linoleic acid
LDL-C	low-density lipoprotein cholesterol
PSM	perilla seed meal
PUFA	polyunsaturated fatty acid
RSM	rapeseed meal
SEM	standard error of the means
SFA	saturated fatty acids
TG	triglyceride
TME	true metabolizable energy
TP	total protein
UA	uric acid
