# Supplementary material for: Effects of Dietary Inclusion of Perilla Seed Meal on Growth Performance, Plasma Biochemistry, and Breast Muscle Fatty Acid Composition in Sansui Ducks from 4 to 8 Weeks of Age
Source: Animals (Basel). 2026 Mar 10;16(6):860. doi: 10.3390/ani16060860 (PMC13023325; doi:10.3390/ani16060860)
Supplement: Supplementary file 1 [file animals-16-00860-s001.zip › animals-4149795-supplementary.pdf]

Table S1 The amino acids composition of perilla seed meal (as-fed basis)

Essential amino-acid	Content (%)	Non-essential amino-acid	Content (%)
Met	0.902	His	0.961
Lys	1.380	Cys	0.628
Thr	1.215	Gly	1.502
Arg	3.597	Ser	1.600
Ile	1.183	Pro	1.124
Leu	2.159	Ala	1.538
Val	1.563	Asp	2.800
Phe	1.694	Glu	5.181

Table S2 The fatty acids composition of perilla seed meal (as-fed basis)

Fatty acids (μg/g)	Content	Fatty acids (μg/g)	Content
C6:0	0.66	C18:1 n-7	126.74
C8:0	1.02	C18:2 n-6T	0.21
C10:0	0.14	C18:2 n-6 (LA)	2071.86
C11:0	0.54	C20:0	22.21
C12:0	0.67	C18:3 n-6 (GLA)	39.67
C13:0	0.77	C20:1	3510.09
C14:0	3.47	C18:3 n-3 (ALA)	8750.80
C15:0	2.07	C21:0	2.07
C16:0	785.04	C20:2	5.44
C16:1	21.00	C22:0	5.21
C17:0	6.63	C20:3 n-6 (DGLA)	3.61
C17:1T	0.72	C20:3 n-3	8.80
C18:0	222.20	C20:4 n-6 (AA)	4.50
C18:1 n-9T	3.17	C23:0	3.46
C18:1 n-12	1181.75	C20:5 n-3 (EPA)	2.66
C18:1 n-9	791.89	C24:0	9.45

Table S3 The amino acid digestibility of perilla seed meal on Sansui ducks by emptying-force feeding method. [1]

Amino acids	Apparent digestibility (%)	True digestibility (%)
Met	93.24±3.61	95.33±3.61
Cys	87.82±5.61	90.82±5.61
Met+Cys	91.02±4.31	92.25±4.31
Lys	87.54±4.33	88.91±4.33
Thr	87.59±5.24	89.14±5.24
Arg	96.08±1.59	96.61±1.59
Ile	87.75±5.89	89.34±5.89
Leu	89.76±4.37	90.63±4.37
Val	85.89±6.56	87.1±6.56
His	93.68±2.41	95.63±2.41
Phe	92.18±3.32	93.29±3.32
Gly	85.10±7.14	86.36±7.14

Ser	89.97±4.76	91.14±4.76
Pro	86.18±7.18	87.85±7.18
Ala	82.35±8.45	83.57±8.45
Asp	89.29±4.84	89.97±4.84
Glu	89.84±5.91	90.20±5.91

[1] Values are mean of 6 replicates.
